# Breastfeeding and complementary feeding associated with body composition in 18–19 years old adolescents in the 1993 Pelotas Birth Cohort

**DOI:** 10.1186/s40795-017-0201-z

**Published:** 2017-12-01

**Authors:** Silvana Paiva Orlandi, David A. González-Chica, Romina Buffarini, Maria Cristina Gonzalez, Ana Maria Baptista Menezes, Fernando C. Barros, Maria Cecília Formoso Assunção

**Affiliations:** 1 Post Graduate Program in Epidemiology, Federal University of Pelotas, Marechal Deodoro 1160, 3rd floor, 96020-220, Pelotas, Pelotas, RS Brazil; 2 Adelaide Medical School, NHMRC Centre of Research Excellence to Reduce Inequality in Heart Disease, The University of Adelaide, Corner of North Terrace and George Street Adelaide SA, Adelaide, 5000 Australia; 3 Post Graduate Program in Health and Behavior, Catholic University of Pelotas, Rua Gonçalves Chaves, 373 – sala 411 prédio C, 96015-560. Pelotas, Pelotas, RS Brazil; 4 School of Nutrition, Federal University of Pelotas, Rua Gomes Carneiro, n°1. CEP 96010-610. Pelotas, Pelotas, RS Brazil

**Keywords:** Adolescence, Air displacement plethysmography, Breastfeeding, Complementary feeding, Fat mass index, Fat-free mass index, Infancy

## Abstract

**Background:**

The first years of life are critical for human development, therefore it is important to investigate early factors that may influence the development of body composition later in life. In this study, we aimed to evaluate the association between breastfeeding duration and age of introduction of complementary feeding with body composition at 18–19 years.

**Methods:**

This is a prospective study conducted with adolescents belonging to the 1993 Pelotas Birth Cohort. Duration of breastfeeding, age of introduction of other types of milk and complementary feeding were obtained from a subsample of this cohort periodically monitored during the first year of life. The fat mass (FM) and fat-free mass (FFM) indices were estimated using plethysmography (BOD POD ™). Crude and adjusted analyses were stratified by sex using a linear regression model.

**Results:**

1438 adolescents (694 boys and 744 girls) had complete information on exposures and outcomes.. Among men, the mean FMI and FFMI were 4.0 ± 3.1 kg / m^2^ and 19.0 ± 1.9 kg / m^2^; and among women, 8.0 ± 3.2 kg / m^2^ and 15.5 ± 1.7 kg / m^2^, respectively. Neither breastfeeding duration nor age of introduction of complementary foods was associated with mean FMI in both sexes. Mean FFMI was higher among women who were breastfed for three months or more and among men who were breastfed for six months or more. Women who started the complementary feeding after five months of age had lower mean FFMI in adolescence.

**Conclusion:**

The data suggest that only mean FFM in adolescence is associated with early feeding behaviors.

**Electronic supplementary material:**

The online version of this article (10.1186/s40795-017-0201-z) contains supplementary material, which is available to authorized users.

## Background

Population-based surveys in Brazil have shown increasing rates of overweight and obesity in all population groups including children and adolescents [1] .

Obesity in adolescents can be assessed using sex and age-specific body mass index (BMI) [2], however, the accuracy of this measure has been questioned because it does not distinguish between fat mass (FM) and fat-free mass (FFM) [3].

Studies on early origins of diseases have identified early life determinants of excess body fat accumulation [4, 5]. The first years of life are critical periods of development. It is thus necessary to better understand early life determinants of diseases as they may have a key role in preventing obesity and several other chronic diseases in adolescents and adults [6]. Recent research has established an association between body composition in adulthood and maternal conditions [7], breastfeeding duration [8–10], weight gain in the first years of life [11–13], and physical activity levels [14–16].

The association of introduction of complementary foods and body composition in childhood was assessed in some studies [17–19] . Although there is scant evidence regarding introduction of complementary foods and body composition on later periods such adolescence and adulthood based on prospective studies [20]. Furthermore, a systematic review on nutritional determinants of fat-free mass in early adulthood showed that most of the studies on body composition used BMI or doubly indirect methods to assess body composition, such as prediction equations based on indirect methods [20].

The aim of this study was to examine the relationship between total duration of breastfeeding and age of introduction of complementary foods during the first months of life and body composition (FM and FFM assessed by plethysmography) in adolescents 18–19 years of age from the 1993 Pelotas Birth Cohort. We expected that those breastefed for longer time and those who have introduced complementary food later will have higher FFMI.

## Methods

### Study subjects

A prospective longitudinal study was conducted with adolescents belonging to the 1993 Pelotas Birth Cohort. The city of Pelotas is located in southern Brazil and had a population of approximately 328,000 inhabitants in 2010 [21].This cohort consisted of children born in 1993 to mothers living in the urban area. Based on the high percentage of hospital deliveries in the city (99% of deliveries), all the five city’s hospitals were daily visited by trained fieldwork team members. Of 5265 mothers who delivered in a hospital in 1993, 5249 were included in the study. Since birth, the cohort members were evaluated on several occasions. At 6, 12 and 48 months, the follows-up were carried out in a subsample which comprised all newborns with low birth weight (LBW) (<2500 g) in addition to a systematic sampling of 20% of the remaining cohort participants. A detailed description of the Pelotas Birth Cohort methods has been published elsewhere [22].This study included participant with information from five follow-ups: at birth, at ages 6–12- and 48-months, and at 18-years.

### Exposures

Early independent variables were prospectively collected using semi-structured questions during the first year of life through household interviews with the mothers. For this analysis, we studied total duration of breastfeeding (whether exclusive, predominant (breast milk is the predominant source of nourisment) or partial (breast milk along with formula), age of introduction of milk products (cow’s milk or infant formula), and age of introduction of complementary foods (fruits, vegetables and/or other solid or semisolid foods). Exclusive breastfeeding was not assessed due to lack of policies to encourage breastfeeding at that time [23] . Furthermore, the median duration of exclusive breastfeeding in our cohort was 0.1 months [24].. We applied two different breastfeeding variables, one in a dichotomous way (yes/no) and the second one in a polytomous way, categorized according to the duration of breastfeeding as never, 0.01–1.00; 1.01–3.00; 3.01–6.00; 6.01–12.00; >12.00 months. The age of introduction of other milk products and complementary foods was categorized as ≤1.00; 1.01–2.00; 2.01–3.00; 3.01–4.00; 4.01–5.00; >5.00 months.

### Outcome measure

The outcome measure in this study was body composition at age 18–19 years measured using air displacement plethysmography (BODPOD®, Life Measurement, Inc., Concord, CA, USA). Plethysmography is a safe, fast, and non-invasive method that can be applied in different population groups (obese individuals, children and the elderly). Therefore it has replaced hydrostatic weighing techniques as the method of choice for the measurement of body density [25]. We excluded participants who were unable to undergo this measure due to physical disabilities and limb amputations, in addition to pregnant and potentially pregnant adolescents and those who gave birth in the 3 months preceding the study (*n* = 68).

The dependent variables were the fat mass index (FMI) and fat-free mass index (FFMI). They were adjusted for body size by dividing FM and FFM by height in meters squared (kg/m^2^) and analyzed as continuous variables. Trained evaluators performed all BODPOD measurements and height measurements taken following a standard protocol using a portable stadiometer accurate to 0.1 cm [26, 27].

### Statistical analyses

In the analysis of this study, cohort participants with complete information on exposures (duration of breastfeeding and age of introduction of other milks and semisolid and solid complementary foods during the first year of life) and outcomes (FMI and FFMI) (*n* = 1438) were included.

For control of potential confounders, the following variables collected in the first follow-up (1993) were studied [20]: maternal age (years); mother’s self-reported skin color (white, black/brown, other); per capita household income (divided into quintiles); maternal education (years of complete schooling); pre-pregnancy BMI (weight/height squared using the mother’s prepregnancy weight and height self-reported in the perinatal interview included as a numeric variable); smoking during pregnancy (yes/no); gestational age (estimated using the last menstrual period; categorized as <37 weeks or ≥37 weeks); and LBW (birth weight < 2500 g).

The analyses were performed using Stata 12.1 (Stata Corp., College Station, Texas, USA). Data was analysed separately by gender as this is a potential effect modifier in the associations investigated due to differences in body composition between males and females [28]. Categorical variables were described as absolute and relative frequencies, and numerical variables as means and standard deviations (median and interquartile range for asymmetric variables). For crude analyses of exposures and outcomes, we used t-test and ANOVA (heterogeneity of variance or trend depending on the exposure variable) and simple linear regression. We conducted analyses with adjustment for potential confounders for each main exposure studied. Potential confounding variables were included in fully adjusted regression models regardless of their level of statistical significance in the bivariate analysis of the association with the outcome In view of evidence showing that many variables can modify the association between exposure variables and outcome measures [20], we carried out analyses of interactions including the variables maternal nutritional status, skin color, education and household income. We performed all crude and adjusted analyses using sampling weights to take into account the sampling selection procedure. Because the distribution of FMI was non-normal, analyses used both the original FMI and the natural-log transformed FMI (lnFMI) to allow comparisons of the results.

Given that the present study had a fixed sample size, we retrospectively calculated the minimum detectable difference using the data available with an alpha error of 5%, power of 80% and FFMI and lnFMI standard deviations. For these estimates, we used the age three months as a cutoff to set the proportion of exposed and non-exposed to the variables breastfeeding and introduction of milk products and complementary foods. Based on these parameters the study could detect mean differences of FFMI between 0.47 and 0.62 in males and 0.39 and 0.53 in females. For lnFMI, the study would have sufficient power to detect differences between 15.6% and 20.6% of males and 9.6% and 14.0% in females.

The Ethics Committee of the Medicine School of Federal University of Pelotas approved all follow-ups of children from the 1993 Pelotas Birth Cohort. All participants or their guardians provided a signed informed consent at all stages of this study.

## Results

A sample of 4106 adolescents aged 18–19 years were examined between September 2011 and March 2012, accounting for 78.2% of the original cohort. Table 1 shows that there were no differences regarding gender, maternal age, skin color and household income between participants of the original cohort and those followed up. However, the attrition rate was higher among children of women at extreme education categories (0–4 years and 12 years or more) and those with a previous LBW baby. As for the 1143 participants in the subsample assessed at ages 6 and 12 months (*n* = 1438), the follow-up rate was 79.0%, with no differences for any of the variables mentioned above compared to the initial subsample.

**Table 1:** Comparison between adolescents who were followed-up at 18-y with the original cohort and the sub-sample at 6 and 12-months according to sociodemographic and anthropometric variables. 1993 Pelotas Birth Cohort

Variable	N original cohort	Cohort members followed in 2011–2012		Sub-sample cohort members (*n* = 1438) followed at 6 and 12 months and located in 2011–2012	
		% lost to follow-up^a^	*p* ^b^	% lost to follow-up^c^	*p* ^d^
All sample	5248	21.8		21.0	
Sex					
Men	2603	22.6	0.149	20.4	0.627
Women	2645	20.9		21.6	
Maternal age (years)					
< 20	915	23.3	0.420	27.3	0.068
20–34	3756	21.6		19.7	
> 35	577	20.6		21.2	
Maternal skin colour ^e^					
White	4058	22.2	0.313	21.9	0.246
Black	955	20.1		18.8	
Others	234	20.5		14.2	
Family income at birth (quintiles) ^e^					
1°	1031	24.4	0.06	24.5	0.502
2°	1195	21.1		21.1	
3°	889	21.0		19.5	
4°	1001	18.9		17.9	
5°	1021	21.9		20.3	
Maternal education (years) ^e^					
0–4	1427	25.5	<0.001	23.4	0.572
5–8	2424	19.1		19.8	
9–11	923	20.8		19.9	
> =12	427	26.2		22.9	
Birth weight (grams) ^e^					
< 2500	510	27.6	0.001	21.2	0.285
≥ 2500	4722	21.0		18.7	

^a^Number of lost to follow-up in 2012 as a percentage of those evaluated in the original cohort. ^b^Chi-squared test of the comparison between the cohort members followed-up in 2012 and the original cohort. ^c^Number of lost to follow-up in 2012 at 6 and 12 months with complete data at 2011–2012 follow-up as a percentage of those evaluated in the sub-sample at 6 and 12 months. ^d^Chi-squared test of the comparison between the cohort members with complete information for analyses at 18 years in relation with the sub-sample followed-up at 6 and 12 months. ^e^Variables with missing data (maximum valor of missing information is 2% for family income)

The proportion of males in the sample was 51.7%. They showed higher FFMI and lower FMI compared to females: mean 19.0 ± 1.9 kg/m^2^ and 4.00 ± 3.1 kg/m^2^ in males and 15.5 ± 1.7 kg/m^2^ and 8.00 ± 3.7 kg/m^2^ in females (*p* < 0.001 for both outcome measures). The median FMI and interquartile ranges were 3.0 (2.0, 5.0) in males and 7.1 (5.5, 9.6) in females (p < 0.001).

In males, FMI was higher among those from families with higher household income (mean FMI 4.6 in the highest quintile vs 3.0 in the lowest quintile, p < 0.001) and was directly associated with pre-pregnancy BMI (mean FMI 6.1 for pre-pregnancy obesity vs 2.8 for pre-pregnancy underweight, p < 0.001). Pre-pregnancy BMI also showed an association with FFMI (mean FFMI 20.4 for pre-pregnancy obesity vs 17.9 for pre-pregnancy underweight, *p* < 0.001). None of the other variables investigated was associated with either FMI or FFMI in males. Female participants born to smoking mothers showed higher FFMI compared with those born to mothers that did not smoke (15.8 vs. 15.4) and those with LBW had higher FMI compared to normal weight (8.3 vs. 7.5). In addition, those born to mothers with black skin color showed lower FMI and higher FFMI at age 18 compared with white and other color. Similarly to that observed in males, pre-pregnancy BMI was positive associated with both FMI (mean FMI 11.0 for pre-pregnancy obesity vs 6.1 for pre-pregnancy underweight, *p* < 0.001) and FFMI (mean FFMI 16.6 for pre-pregnancy obesity vs 14.8 for pre-pregnancy underweight, p < 0.001) **(**Additional file 1: Table S1).

Table 2 shows the associations between nutritional variables and FMI and FFMI. In males, the total breastfeeding (in months) was associated with FFMI (*p* = 0.011). In females, those ever breastfed showed higher FFMI than those never breastfed (*p* = 0.02), and those who were given complementary foods at ages 4–5 months showed greater FFMI and FMI. The median (IQR) of FMI according to the exposures is in Additional file 2: Table S2.

**Table 2:** Descriptive analyses of fat mass index and fat-free mass index at 18 years according to breastfeeding and introduction of complementary feeding during the first year of life, stratified by sex. 1993 Pelotas Birth Cohort (n = 1438)

Independent variables	Men (*n* = 694) Mean (SD)			Women (*n* = 744) Mean (SD)		
	%	FMI	FFMI	%	FMI	FFMI
Total breastfeeding (months)		0.773^a^	0.011^a^		0.528 ^a^	0.063 ^a^
Never	4.1	3.9 (3.2)	18.6 (1.7)	3.5	8.2 (3.2)	14.6 (1.6)
0.01–1.00	17.6	4.3 (3.5)	19.4 (2.1)	15.4	8.2 (3.9)	15.2 (1.7)
1.01–3.00	32.0	3.9 (2.9)	18.9 (1.7)	30.6	7.9 (3.2)	15.5 (1.7)
3.01–6.00	18.5	3.8 (2.7)	18.7 (1.9)	16.0	8.5 (4.1)	15.5 (1.7)
6.01–12.00	12.5	4.4 (3.1)	19.6 (1.9)	12.4	9.1 (5.2)	15.8 (2.0)
> 12.00	15.3	4.3 (3.7)	19.4 (2.1)	22.1	7.9 (3.1)	15.7 (1.5)
Breastfeeding		0.703 ^b^	0.172^b^		0.975 ^b^	0.021 ^b^
No	4.0	3.9 (3.2)	18.6 (1.7)	3.4	8.2 (3.2)	14.6 (1.6)
Yes	96.0	4.1 (3.1)	19.1 (1.9)	96.6	8.2 (3.8)	15.5 (1.6)
Age of introduction of other milks ^c^ (months)		0.175 ^a^	0.187 ^a^		0.188 ^a^	0.624 ^a^
< = 1.00	41.2	4.2 (3.3)	19.3 (2.0)	38.3	8.6 (3.9)	15.4 (1.7)
1.01–2.00	24.4	3.6 (2.4)	18.8 (1.9)	25.3	7.7 (3.1)	15.5 (1.7)
2.01–3.00	14.0	4.1 (3.3)	19.0 (1.7)	16.3	8.4 (4.6)	15.4 (1.8)
3.01–4.00	8.9	3.6 (2.4)	18.7 (1.7)	10.5	8.6 (4.2)	15.7 (1.7)
4.01–5.00	6.8	4.5 (2.7)	19.0 (1.9)	4.9	8.4 (3.5)	15.9 (1.6)
> 5.00	4.7	5.0 (3.3)	19.6 (2.4)	4.8	7.3 (2.8)	15.2 (1.3)
Age of introduction of other foods ^d^ (months)		0.887 ^a^	0.280 ^a^		0.031 ^a^	0.013 ^a^
< = 1.00	3.5	3.5 (3.6)	18.6 (1.4)	5.9	8.5 (2.8)	15.5 (1.4)
1.01–2.00	16.8	4.2 (3.3)	19.3 (2.3)	15.6	8.4 (3.9)	15.5 (1.8)
2.01–3.00	41.2	4.0 (3.0)	19.0 (1.8)	43.1	8.1 (3.7)	15.5 (1.6)
3.01–4.00	27.7	3.9 (2.9)	19.1 (1.9)	23.4	7.7 (3.4)	15.5 (1.6)
4.01–5.00	8.5	4.4 (3.1)	19.9 (2.1)	9.7	9.2 (4.7)	15.8 (2.0)
> 5.00	2.4	4.6 (4.0)	18.1 (2.0)	2.3	6.6 (2.1)	14.5 (1.1)

Abbreviations: *SD* standard deviation, *FMI* fat mass index, *FFMI* fat-free mass index

^a^Test for Heterogeneity. ^b^T test. ^c^ Cow milk and formula. ^d^Fruits, vegetables and others

Table 3 presents crude and adjusted coefficients of the association of breastfeeding and age of introduction of complementary foods with FMI at age 18–19 years. There were no statistically significant associations between duration of breastfeeding or age of introduction of other milk products and FMI. Regarding the age of introduction of complementary foods, the crude analysis showed lower FMI among females who were given complementary foods after the age of five months. However, these differences lost statistical significance after adjustment for confounders. We repeated these analyses using lnFMI, but there were no changes either in the direction of the association or the statistical significance **(**Additional file 3: Table S3).

**Table 3:** Crude and adjusted analyses for fat mass index at 18 years according to breastfeeding and introduction of complementary feeding during the first year of life, stratified by sex. 1993 Pelotas Birth Cohort

Independent variables	Male				Female			
	Crude		Adjusted^a^		Crude		Adjusted^a^	
	β	CI95%	Β	CI95%	Β	CI95%	β	CI95%
Total breastfeeding (months)		0.773 ^a^		0.707 ^a^		0.528 ^a^		0.171 ^a^
Never	Ref. (0)		Ref. (0)		Ref. (0)		Ref. (0)	
0.01–1.00	0.45	−1.01, 1.92	0.90	−0.63, 2.44	0.06	−1.64, 1.76	−0.14	−1,89, 1.62
1.01–3.00	0.07	−1.26, 1.40	0.23	−1.19, 1.65	−0.29	−1.83, 1.24	−0.89	−2.49, 0.73
3.01–6.00	−0.05	−1.42, 1.32	0.29	−1.18, 1.75	−0.32	−1.40, 2.03	0.14	−1.61, 1.88
6.01–12.00	0.51	−0.95, 1.97	0.55	−1.04, 2.13	0.91	−1.02, 2.85	0.60	−1.41, 2.61
> 12.00	0.46	−1.05, 1.98	0.62	−0.94, 2.19	−0.29	−1.85, 1.26	−0.58	−2.20, 1.04
Breastfeeding		0.703 ^b^		0.471 ^b^		0.975 ^a^		0.788 ^a^
No	Ref. (0)		Ref. (0)		Ref. (0)		Ref. (0)	Ref. (0)
Yes	0.25	−1.03, 1.52	0.50	−0.86, 1.87	0.02	−1.46, 1.50	−0.21	−1.78, 1.35
Age of introduction of other milks ^c^ (months)		0.175 ^a^		0.371 ^a^		0.188 ^a^		0.277 ^a^
< = 1.00	Ref. (0)		Ref. (0)		Ref. (0)		Ref. (0)	
1.01–2.00	−0.55	−1.24, 0.14	−0.48	−1.17, 0.21	−0.90	−1.77, −0.03	−0.77	−1.65, 0.10
2.01–3.00	−0.07	−1.05, 0.90	0.06	−0.89, 1.01	−0.20	−1.45, 1.06	0.05	−1.18, 1.28
3.01–4.00	−0.59	−1.49, 0.31	−0.40	−1.30, 0.49	0.02	−1.37, 1.42	0.59	−0.75, 1.93
4.01–5.00	0.31	−0.84, 1.46	0.09	−1.04, 1.22	−0.18	−1.84, 1.47	0.07	−1.46, 1.59
> 5.00	0.89	−0.44, 2.22	0.79	−0.53, 2.11	−1.27	−2.46, −0.08	−0.57	−1.60, 0.46
Age of introduction of other foods ^d^ (months)		0.887 ^a^		0.561 ^a^		0.031 ^a^		0.176 ^a^
< = 1.00	Ref. (0)		Ref. (0)		Ref. (0)		Ref. (0)	
1.01–2.00	0.67	−1.33, 2.67	0.68	−1.28, 2.63	−0.17	−1.60, 1.26	0.18	−1.10, 1.46
2.01–3.00	0.50	−1.41, 2.42	0.16	−1.72, 2.04	−0.43	−1.67, 0.81	−0.17	−1.23, 0.90
3.01–4.00	0.38	−1.56, 2.32	−0.03	−1.93, 1.88	−0.85	−2.14, 0.45	−0.31	−1.45, 0.83
4.01–5.00	0.89	−1.19, 2.98	0.59	−1.41, 2.59	0.61	−1.16, 2.39	0.86	−0.68, 2.41
> 5.00	1.10	−1.57, 3.77	1.04	−1.55, 3.64	−1.99	−3.55, −0.43	−1.13	−2.51, 0.24

Abbreviations: *β* regression coefficient, *CI 95%* 95% confidence interval. ^a^Test for heterogeneity T test. ^b^T test. ^c^Cow milk and formula. ^d^Fruits and vegetables and others

Adjusted for maternal age, maternal skin color, maternal schooling, family income, maternal prepregnancy body mass index, maternal smoking during pregnancy, low birth weight and gestational age

On the other hand, FFMI was significantly associated with breastfeeding and age of introduction of complementary foods as shown in Table 4. Regarding the duration of breastfeeding, there was a positive association of breastfeeding on FFMI. Higher FFMI was found in males in both crudes and adjusted analyses, and the breastfeeding for six to 12 months of age showed the greatest mean difference compared to the reference category (β = 1.20; 95%CI 0.39–2.01), followed by the breastfeeding longer than 12 months. It was also observed a positive effect of breastfeeding on FFMI in females, though it was on the borderline of significance in the crude and adjusted analyses. The same is true when considering the 95% CI, which is statistically different from the reference category starting from breastfeeding longer than three to six months (β = 0.98; 95%CI 0.18–1.77). This difference remains the same as the duration of breastfeeding increases. When we assessed the association of breastfeeding as a dichotomous variable, the positive effect on FFMI was not evidenced in males (borderline effect in the adjusted analysis) while it became statistically significant in females. Age of introduction of complementary foods was the single variable associated with FFMI; lower FFMI was seen among females who were given complementary foods after five months of age (*p* = 0.036).

**Table 4:** Crude and adjusted analyses for fat-free mass index at 18 years according to breastfeeding and introduction of complementary feeding during the first year of life, stratified by sex. 1993 Pelotas Birth Cohort

Independent variables	Male				Female			
	Crude		Adjusted^a^		Crude		Adjusted^a^	
	β	IC95%	β	IC95%	β	IC95%	β	IC95%
Total breastfeeding (months)		0.011 ^a^		0.006 ^a^		0.063 ^a^		0.079 ^a^
Never	Ref. (0)		Ref. (0)		Ref. (0)		Ref. (0)	
0.01–1.00	0.79	−0.01, 1.58	0.92	0.08, 1.75	0.60	−0.26, 1.47	0.53	−0.26, 1.32
1.01–3.00	0.31	−0.39, 1.00	0.37	−0.38, 1.11	0.89	0.06, 1.72	0.63	−0.13, 1.39
3.01–6.00	0.04	−0.71, 0.79	0.29	−0.51, 1.08	0.95	0.08, 1.82	0.98	0.18, 1.77
6.01–12.00	0.99	0.18, 1.79	1.20	0.39, 2.01	1.44	0.27, 2.12	1.01	0.13, 1.89
> 12.00	0.75	−0.06, 1.56	0.89	0.04, 1.73	1.09	0.26, 1.92	0.90	0.13, 1.67
Breastfeeding		0.172 ^b^		0.083 ^b^		0.021 ^a^		0.035 ^a^
No	Ref. (0)		Ref. (0)		Ref. (0)		Ref. (0)	
Yes	0.46	−0.20, 1.12	0.63	−0.08, 1.34	0.94	0.14, 1.73	0.79	0.06, 1.52
Age of introduction of other milks ^c^(months)		0.187 ^a^		0.368 ^a^		0.625 ^a^		0.131 ^a^
< = 1.00	Ref. (0)		Ref. (0)		Ref. (0)		Ref. (0)	
1.01–2.00	−0.52	−0.98, −0.05	−0.40	−0.88, 0.08	0.07	−0.36, 0.50	0.24	−0.17, 0.66
2.01–3.00	−0.34	−0.86, 0.19	−0.08	−0.59, 0.44	0.02	−0.49, 0.54	0.10	−0.40, 0.60
3.01–4.00	−0.59	−1.20, 0.02	−0.39	−0.95, 0.17	0.29	−0.26, 0.85	0.60	0.05, 1.15
4.01–5.00	−0.36	−1.15, 0.43	−0.09	−0.84, 0.67	0.52	−0.23, 1.28	0.73	0.07, 1.39
> 5.00	0.30	−0.84, 1.45	0.57	−0.55, 1.68	−0.18	−0.78, 0.42	0.01	−0.53, 0.55
Age of introduction of other foods ^d^ (months)		0.280 ^a^		0.493 ^a^		0.014 ^a^		0.036 ^a^
< = 1.00	Ref. (0)		Ref. (0)		Ref. (0)		Ref. (0)	
1.01–2.00	0.74	−0.12, 1.60	0.66	−0.14, 1.46	−0.08	−0.79, 0.62	0.09	−0.69, 0.51
2.01–3.00	0.44	−0.31, 1.20	0.36	−0.34, 1.05	−0.03	−0.66, 0.59	−0.11	−0.62, 0.40
3.01–4.00	0.58	−0.21, 1.37	0.55	−0.17, 1.28	−0.09	−0.74, 0.56	0.02	−0.53, 0.56
4.01–5.00	0.46	−0.49, 1.41	0.43	−0.41, 1.27	0.24	−0.59, 1.06	0.31	−0.36, 0.98
> 5.00	−0.43	−1.67, 0.81	−0.13	−1.44, 1.18	−1.07	−1.85, −0.28	−0.77	−1.43, −0.11

Abbreviations: *β* regression coefficient, *CI 95%* 95% confidence interval

Adjusted for maternal age, maternal skin color, maternal schooling, family income, maternal prepregnancy body mass index, maternal smoking during pregnancy, low birth weight and gestational age

^a^Test for heterogeneity. ^b^T test. ^c^Cow milk and formula. ^d^Fruits and vegetables and others

The interaction analyses including the variables maternal nutritional status, skin color, education, and household income showed no effect modification on the associations.

## Discussion

Studies of early life origins of diseases have suggested that adult health-disease programming can occur as an effect of early exposures during the first years of life [6]. Many studies have investigated the effect of feeding during the first year of life on later life outcomes including a protective effect of breastfeeding against child and adolescent obesity and development of chronic diseases such as hypertension and diabetes mellitus [9]. Strengths of this study include its prospective nature, which permitted the analysis of the effect of breastfeeding and age of introduction of milk products and complementary foods (solid and semisolid foods) on body composition in late adolescence.

Also, body composition was assessed using an accurate assessment method [25, 26], with body fat being measured with its compartments (FM and FFM). Another key aspect of this study was the age at which the outcome was evaluated as most studies published to date have assessed body composition in children. The assessment of body composition in late adolescence, a period that is characterized by rapid physical changes with marked effects on adult body composition [13], allows to investigate whether the potential effects of early nutrition are extended beyond childhood.

After adjusting for potential confounders, this study found no statistically significant association between breastfeeding and age of introduction of milk products or complementary foods with FMI at age 18. Victora et al. reported similar results while studying participants from another Pelotas birth cohort. They assessed 2250 young adult males 18 years of age and found no association between duration of breastfeeding and adiposity (FM and FMI) measured by bioelectrical impedance [29].

In contrast, one prospective cohort study conducted in the United Kingdom with 536 children born in 1988 found breastfeeding duration to be associated with FM (measured by dual-energy X-ray absorptiometry) after comparing children breastfed for 12 months or more with those who were never breastfed. Those never breastfed had higher FM at age four years (*p* = 0.002). However, it should be noted these are short-term results and also prone to residual confounding and reverse casuality because of observational design [30].

Studies on early introduction of complementary foods and FFM are scarce and inconclusive [20]. In our study, we found associations of both breastfeeding and age of introduction of complementary foods with FFMI.

Although the World Health Organization recommends exclusive breastfeeding for the first six months of life [31], in our study we found that introduction of complementary foods (fruits, vegetables, solid and semi-solid foods) before six months of age was associated with higher FFMI among girls, which has positive implications for health.=. However, caution must be applied as our study did not evaluate the types of complementary foods introduced before six months of age. While there is well-established evidence of short-term benefits of breastfeeding, particularly on the reduction of child morbidity and mortality from infectious diseases [32], long-term consequences have only recently been studied. Horta et al. conducted a meta-analysis of 71 studies on the impact of breastfeeding and nutritional status at later ages and found a protective effect of breastfeeding against overweight/obesity in later life [9].

In women we found evidence of increased FFMI with a shorter time of exposure of breastfeeding and earlier introduction of complementary foods (semisolid and solid) when compared to men. This gender difference pointing to a female benefit in terms of body composition (e.g. increased FFMI) is biologically plausible since female infants are born with a higher physiological maturity in relation to male [28, 33, 34]. For this reason, we hypothesized that female infants would benefit more of introducing nutrients from semisolid and solid foods at an earlier age in relation to boys.

In a recent meta-analysis Horta et al. verified that the larger the size of the sample of studies evaluating the effect of breastfeeding on adult overweight and/or obesity, the less evident the impact of this association [9]. Although the loss to follow-up was about one-third of the sample in our study, it was not differential by either sociodemographic or birth characteristics.

## Conclusion

In conclusion, this study found significant associations between early nutritional practices and long-term effects on FFMI. It may suggest that both breastfeeding (in male and female infants) and age of introduction of complementary foods (in female infants only) independently affect body composition in young adults.

## Additional files

Additional file 1: Descriptive analyses of fat mass index and fat-free mass index at 18 years according to sociodemographic and anthropometric variables at birth, stratified by sex. 1993 Pelotas Birth Cohort (*n* = 1438) (DOCX 31 kb)

Additional file 2: Descriptive analyses of median (IQR) of fat mass index at 18 years according to breastfeeding and introduction of complementary feeding during the first year of life, stratified by sex. 1993 Pelotas Birth Cohort (n = 1438) (DOCX 29 kb)

Additional file 3: Crude and adjusted analyses for the log-transformed fat mass index at 18 years according to breastfeeding and introduction of complementary feeding during the first year of life, stratified by sex. 1993 Pelotas Birth Cohort (DOCX 17 kb)

## Abbreviations

FM: Fat mass
FFM: Fat-free mass
BOD POD ™: Plethysmography
BMI: Body mass index
FMI: Fat mass index
FFMI: Fat-free mass index
lnFMI: Natural-log transformed FMI
LBW: Low birth weight
