# Supplementary material for: Breastfeeding and complementary feeding associated with body composition in 18–19 years old adolescents in the 1993 Pelotas Birth Cohort
Source: BMC Nutr. 2017 Dec 1;3:84. doi: 10.1186/s40795-017-0201-z (PMC7050824; doi:10.1186/s40795-017-0201-z)
Supplement: Supplementary file 1 — Descriptive analyses of fat mass index and fat-free mass index at 18 years according to sociodemographic and anthropometric variables at birth, stratified by sex. 1993 Pelotas Birth Cohort (n = 1438) (DOCX 31 kb) [file 40795_2017_201_MOESM1_ESM.docx]

Additional file 1. Descriptive analyses of fat mass index and fat-free mass index at 18 years according to sociodemographic and anthropometric variables at birth, stratified by sex. 1993 Pelotas Birth Cohort (n=1438).

| Variables | Male (694)  Mean (SD) | | | Female (744)  Mean (SD) | | |
| --- | --- | --- | --- | --- | --- | --- |
|  | % | FMI | FFMI | % | FMI | FFMI |
| Maternal age (years) |  | 0.618^a^ | 0.806^b^ |  | 0.935^b^ | 0.709^b^ |
| < 20 | 15.4 | 4.2 (2.8) | 19.2 (2.1) | 15.3 | 8.2 (4.6) | 15.6 (1.9) |
| 20 – 34 | 73.7 | 4.1 (3.1) | 19.0 (1.9) | 73.7 | 8.1 (3.5) | 15.5 (1.7) |
| >= 35 | 10.9 | 3.9 (3.1) | 19.2 (1.7) | 10.9 | 8.4 (4.2) | 15.6 (1.6) |
| Maternal skin colour |  | 0.528^b^ | 0.138^b^ |  | 0.014^b^ | 0.005^b^ |
| White | 80.2 | 4.1 (3.1) | 19.0 (1.9) | 77.0 | 8.4 (3.9) | 15.4 (1.7) |
| Black | 14.4 | 4.2 (3.3) | 19.3 (2.1) | 18.3 | 7.3 (3.2) | 16.0 (1.6) |
| Others | 5.4 | 3.6 (2.4) | 19.5 (1.8) | 4.7 | 8.1 (3.7) | 15.3 (1.6) |
| Family income (quintiles) |  | < 0.001^b^ | 0.658^b^ |  | 0.725^b^ | 0.752^b^ |
| 1° | 20.6 | 3.0 (2.3) | 18.9 (1.7) | 19.3 | 7.8 (3.9) | 15.7 (1.9) |
| 2° | 25.3 | 4.3 (3.4) | 19.2 (1.9) | 23.3 | 8.1 (3.7) | 15.5 (1.7) |
| 3° | 17.4 | 4.1 (2.9) | 19.1 (2.1) | 19.6 | 8.1 (3.6) | 15.3 (1.5) |
| 4° | 17.5 | 4.5 (3.4) | 18.9 (2.1) | 17.4 | 8,6 (4.2) | 15.6 (1.7) |
| 5° | 19.2 | 4.6 (3.2) | 19.1 (2.0) | 20.3 | 8.2 (3,5) | 15.5 (1.6) |
| Maternal schooling (years) |  | 0.058 ^a^ | 0.534^b^ |  | 0.590^b^ | 0.310^b^ |
| 0 – 4 | 24.4 | 3.6 (3.0) | 19.2 (2.1) | 29.3 | 8.4 (4.0) | 15.7 (1.8) |
| 5 – 8 | 49.2 | 4.2 (3.1) | 19.1 (1.9) | 45.0 | 8.0 (3.6) | 15.4 (1.7) |
| 9 – 11 | 17.1 | 4.2 (3.1) | 18.8 (1.7) | 18.2 | 8.5 (3.9) | 15.4 (1.5) |
| > =12 | 9.2 | 4.7 (3.1) | 19.2 (2.3) | 7.5 | 7.9 (3.5) | 15.7 (1.5) |
| Maternal prepregnancy BMI |  | <0.001 ^a^ | <0.001 ^a^ |  | <0.001 ^a^ | <0.001 ^a^ |
| Underweight | 10.0 | 2.8 (2.3) | 17.9 (1.7) | 7.7 | 6.1 (2.1) | 14.8 (1.5) |
| Normal | 68.9 | 4.1 (3.0) | 19.0 (1.8) | 70.3 | 7.9 (3.7) | 15.4 (1.6) |
| Overweight | 14.6 | 4.2 (2.9) | 19.3 (2.0) | 16.6 | 9.6 (4.0) | 16.2 (1.8) |
| Obesity | 6.5 | 6.1 (4.5) | 20.4 (2.3) | 5.2 | 11.0 (3.7) | 16.6 (1.7) |
| Maternal smoking during pregnancy |  | 0.672^c^ | 0.450 ^c^ |  | 0.178 ^c^ | 0.008 ^c^ |
| Yes | 32.5 | 4.0 (3.1) | 19.2 (2.0) | 33.7 | 8.5 (4.0) | 15.8 (1.7) |
| No | 67.5 | 4.1 (3.1) | 19.0 (1.9) | 66.3 | 8.0 (3.6) | 15.4 (1.7) |
| Low birth weight (grams) |  | 0.101 ^c^ | 0.550 ^c^ |  | 0.010 ^c^ | 0.224 ^c^ |
| <2500 | 8.1 | 4.1 (3.1) | 19.1 (1.9) | 11.3 | 8.3 (3.8) | 15.5 (1.7) |
| ≥2500 | 91.9 | 3.7 (2.9) | 18.9 (1.9) | 88.7 | 7.5 (3.4) | 15.4 (1.7) |
| Gestational age (weeks) |  | 0.667 ^c^ | 0.918 ^c^ |  | 0.566 ^c^ | 0.349 ^c^ |
| <37 | 7.9 | 4.1 (3.1) | 19.0 (1.9) | 8.9 | 8.2 (3.8) | 15.5 (1.7) |
| ≥37 | 92.1 | 3.9 (2.7) | 19.1 (1.7) | 91.1 | 7.9 (3.4) | 15.3 (1.7) |

Abbreviations: SD, standard deviation; FMI, fat mass index; FFMI. fat-free mass index; BMI, body mass index.

^a^Test for linear trend. ^b^Test for heterogeneity. ^c^T test.
