# Supplementary material for: Breastfeeding and complementary feeding associated with body composition in 18–19 years old adolescents in the 1993 Pelotas Birth Cohort
Source: BMC Nutr. 2017 Dec 1;3:84. doi: 10.1186/s40795-017-0201-z (PMC7050824; doi:10.1186/s40795-017-0201-z)
Supplement: Supplementary file 2 — Descriptive analyses of median (IQR) of fat mass index at 18 years according to breastfeeding and introduction of complementary feeding during the first year of life, stratified by sex. 1993 Pelotas Birth Cohort (n = 1438) (DOCX 29 kb) [file 40795_2017_201_MOESM2_ESM.docx]

Supplementary Material

Descriptive analyses of median (IQR) of fat mass index at 18 years according to breastfeeding and introduction of complementary feeding during the first year of life, stratified by sex. 1993 Pelotas Birth Cohort (n=1438)

| Independent variables | Men (n=694) | | Women (n=744) | |
| --- | --- | --- | --- | --- |
| Total breastfeeding (months) | Median | (IQR)^c^ | Median | (IQR) ^c^ |
| Never |  |  | 7.2 | (3.7) |
| 0.01 – 1.00 | 2.6 | (2.7) | 7.0 | (3.6) |
| 1.01 – 3.00 | 3.0 | (3.6) | 7.0 | (3.4) |
| 3.01 – 6.00 | 2.8 | (2.9) | 6.8 | (4.7) |
| 6.01 – 12.00 | 3.3 | (2.7) | 7.5 | (5.3) |
| > 12.00 | 2.6 | (3.9) | 7.3 | (3.8) |
| Breastfeeding |  |  |  |  |
| No | 2.6 | (2.7) | 7.2 | (3.7) |
| Yes | 3.0 | (3.1) | 7.1 | (4.1) |
| Age of introduction of other milks ^a^ (months) | |  |  |  |
| <= 1.00 | 3.0 | (3.0) | 7.4 | (4.3) |
| 1.01 – 2.00 | 2.9 | (2.6) | 6.5 | (3.7) |
| 2.01 – 3.00 | 3.1 | (3.2) | 6.7 | (4.0) |
| 3.01 – 4.00 | 2.8 | (2.0) | 7.0 | (4.2) |
| 4.01 – 5.00 | 3.7 | (4.3) | 7.6 | (5.4) |
| > 5.00 | 4.7 | (5.3) | 6.6 | (4.3) |
| Age of introduction of other foods ^b^(months) | |  |  |  |
| <= 1.00 | 2.4 | (2.7) | 7.4 | (4.7) |
| 1.01 – 2.00 | 3.0 | (3.3) | 7.4 | (4.5) |
| 2.01 – 3.00 | 3.1 | (3.1) | 7.2 | (3.8) |
| 3.01 – 4.00 | 2.9 | (2.7) | 6.5 | (3.5) |
| 4.01 – 5.00 | 3.1 | (3.9) | 7.3 | (4.9) |
| > 5.00 | 3.5 | (4.1) | 5.3 | (2.7) |

Abbreviations: IQR, interquartile range; FMI, fat mass index;

^a^ Cow milk and formula. ^b^Fruits, vegetables and others.

^c^ IQR: percentile 75 - percentile 25
