# Supplementary material for: Breastfeeding and complementary feeding associated with body composition in 18–19 years old adolescents in the 1993 Pelotas Birth Cohort
Source: BMC Nutr. 2017 Dec 1;3:84. doi: 10.1186/s40795-017-0201-z (PMC7050824; doi:10.1186/s40795-017-0201-z)
Supplement: Supplementary file 3 — Crude and adjusted analyses for the log-transformed fat mass index at 18 years according to breastfeeding and introduction of complementary feeding during the first year of life, stratified by sex. 1993 Pelotas Birth Cohort (DOCX 17 kb) [file 40795_2017_201_MOESM3_ESM.docx]

Additional file 2. Crude and adjusted analyses for the log-transformed fat mass index at 18 years according to breastfeeding and introduction of complementary feeding during the first year of life, stratified by sex. 1993 Pelotas Birth Cohort.

| Independent variables | Male | | | | Female | | | |
| --- | --- | --- | --- | --- | --- | --- | --- | --- |
|  | Crude | | Adjusted | | Crude | | Adjusted | |
|  | RR | 95% CI | RR | 95% CI | RR | 95% CI | RR | 95% CI |
| Total breastfeeding (months) |  | 0.589 ^a^ |  | 0.543 ^a^ |  | 0.838 ^a^ |  | 0.360 ^a^ |
| Never | Ref. (1) |  | Ref. (1) |  | Ref. (1) |  | Ref. (1) |  |
| 0.01 – 1.00 | 1.07 | 0.80, 1.45 | 1.18 | 0.87, 1.61 | 0.98 | 0.81, 1.19 | 0.97 | 0.81, 1.17 |
| 1.01 – 3.00 | 0.98 | 0.73, 1.29 | 1.00 | 0.74, 1.35 | 0.96 | 0.80, 1.14 | 0.90 | 0.75, 1.07 |
| 3.01 – 6.00 | 1.01 | 0.76, 1.33 | 1.10 | 0.81, 1.48 | 1.01 | 0.83, 1.22 | 0.98 | 0.82, 1.19 |
| 6.01 – 12.00 | 1.17 | 0.87, 1.56 | 1.17 | 0.85, 1.60 | 1.04 | 0.85, 1.28 | 1.00 | 0.82, 1.23 |
| > 12.00 | 1.03 | 0.75, 1.40 | 1.07 | 0.78, 1.47 | 0.96 | 0.80, 1.14 | 0.93 | 0.78, 1.11 |
| Breastfeeding |  | 0.791 ^b^ |  | 0.516 ^b^ |  | 0.806 ^b^ |  | 0.579 ^b^ |
| No | Ref. (1) |  | Ref. (1) |  | Ref.(1) |  | Ref.(1) |  |
| Yes | 1.03 | 0.80, 1.34 | 1.09 | 0.83, 1.44 | 0.98 | 0.83, 1.16 | 0.95 | 0.81, 1.13 |
| Age of introduction of other milks (months) |  | 0.158 ^a^ |  | 0.407 ^a^ |  | 0.362 ^a^ |  | 0.520 ^a^ |
| <= 1.00 | Ref. (1) |  | Ref. (1) |  | Ref. (1) |  | Ref. (1) |  |
| 1.01 – 2.00 | 0.91 | 0.75, 1.10 | 0.91 | 0.75, 1.10 | 0.91 | 0.83, 1.00 | 0.92 | 0.84, 1.01 |
| 2.01 – 3.00 | 0.99 | 0.80, 1.24 | 1.01 | 0.83, 1.24 | 0.95 | 0.84, 1.08 | 0.97 | 0.86, 1.09 |
| 3.01 – 4.00 | 0.93 | 0.75, 1.16 | 0.98 | 0.79, 1.22 | 0.98 | 0.84, 1.14 | 1.03 | 0.89, 1.20 |
| 4.01 – 5.00 | 1.17 | 0.89, 1.54 | 1.06 | 0.82, 1.37 | 0.97 | 0.79, 1.20 | 0.99 | 0.81, 1.20 |
| > 5.00 | 1.34 | 0.99, 1.82 | 1.31 | 0.96, 1.79 | 0.87 | 0.75, 1.02 | 0.94 | 0.83, 1.07 |
| Age of introduction of other foods (months) ^a^ |  | 0.574 ^a^ |  | 0.268 ^a^ |  | 0.062 ^a^ |  | 0.291 ^a^ |
| <= 1.00 | Ref. (1) |  | Ref. (1) |  | Ref. (1) |  | Ref. (1) |  |
| 1.01 – 2.00 | 1.28 | 0.82, 2.02 | 1.29 | 0.82, 2.03 | 0.95 | 0.81, 1.12 | 0.99 | 0.86, 1.15 |
| 2.01 – 3.00 | 1.26 | 0.82, 1.94 | 1.16 | 0.75, 1.80 | 0.92 | 0.80, 1.07 | 0.95 | 0.84, 1.08 |
| 3.01 – 4.00 | 1.23 | 0.79, 1.90 | 1.11 | 0.71, 1.73 | 0.87 | 0.75, 1.02 | 0.92 | 0.81, 1.06 |
| 4.01 – 5.00 | 1.45 | 0.91, 2.29 | 1.35 | 0.86, 2.14 | 1.02 | 0.85, 1.23 | 1.04 | 0.89, 1.23 |
| > 5.00 | 1.42 | 0.82, 2.47 | 1.42 | 0.84, 2.42 | 0.78 | 0.64, 0.96 | 0.86 | 0.71, 1.04 |

Abbreviations: RR, relative risk; 95% CI, 95% confidence interval.

Adjusted for maternal age, maternal skin color, maternal schooling, family income, maternal prepregnancy body mass index, maternal smoking during pregnancy, low birth weight and gestational age.

^a^Test for heterogeneity. ^b^T test. ^c^Cow milk and formula. ^d^Fruits, vegetables and others.
